# The Effects of Hemp Hay (*Canapa sativa* L.) in the Diets of Grazing Goats on Milk Production and Fatty Acid Profile

**DOI:** 10.3390/ani14162373

**Published:** 2024-08-16

**Authors:** Piera Iommelli, Fabio Zicarelli, Ruggero Amato, Nadia Musco, Fiorella Sarubbi, Lucia Bailoni, Pietro Lombardi, Federica Di Bennardo, Federico Infascelli, Raffaella Tudisco

**Affiliations:** 1 Department of Veterinary Medicine and Animal Production, University of Napoli Federico II, 80100 Napoli, NA, Italy; piera.iommelli@unina.it (P.I.); fabiozicarelli@gmail.com (F.Z.); ru.amato@outlook.com (R.A.); pilombar@unina.it (P.L.); dibennardofederica@gmail.com (F.D.B.); infascel@unina.it (F.I.); tudisco@unina.it (R.T.); 2 Institute for the Animal Production System in the Mediterranean Environment, National Research Council, 80055 Portici, NA, Italy; fiorella.sarubbi@cnr.it; 3 Department of Comparative Biomedicine and Food Science (BCA), University of Padova, 35020 Legnaro, PD, Italy; lucia.bailoni@unipd.it

**Keywords:** goat, hemp, milk fatty acid, milk

## Abstract

**Simple Summary:**

Hemp (*Cannabis sativa* L.), traditionally used for textile fiber, has gained interest as animal feed due to its nutritional properties. This study investigated the impact of supplementing goats’ diets with hemp hay on the milk yield, composition, and fatty acid profile of 20 dairy goats. The milk yield was higher in the hemp group, though the milk composition remained unchanged. The fatty acid profile of the hemp group’s milk showed lower levels of certain saturated fatty acids (C11:0, C12:0, C13:0, C14:0, C15:0, and C17:0) and higher levels of C16:0 and C18:0. Additionally, the hemp group’s milk had lower levels of some polyunsaturated fatty acids (C18:2 n6 and C20:4) but higher C20:5 n3 levels. Hemp hay may represent an advantageous alternative as feed supplementation in grazing goats’ diet. Also, in terms of eco-sustainability and the efficiency of agricultural production, the hemp hay is a by-product of the supply chain, which may represent an additional benefit of its use in ruminant nutrition.

**Abstract:**

Hemp (*Cannabis sativa* L.) is a cosmopolitan annual herbaceous plant used in the past as a source of textile fiber. Currently, hemp is receiving great interest as animal feed due to its chemical and nutritional properties. The aim of this study was to explore the effects of supplementing goats’ diets with hemp hay on the milk yield, chemical composition, and fatty acid profile. Twenty multiparous goats, immediately after kidding, were divided into two homogenous groups (C: control vs. H: hemp); the goats had free access to the pasture, and both groups received a supplement of 500 g/head/day of a barley and corn meal mixture (50/50). In addition, group H was given 250 g/head/day of hemp hay while group C received the same amount of alfalfa hay. The milk yield was measured daily, and milk samples were collected monthly 4 times to evaluate the milk composition and fatty acid profile. The milk yield was significantly (*p* < 0.05) higher in the experimental group, while no differences were found in the milk chemical composition. Concerning the fatty acid profile, the milk from group H was characterized by significantly lower concentrations of C11:0, C12:0, C13:0, C14:0, C15:0, and C17:0 and higher C16:0 and C18:0. Among the polyunsaturated FA, C18:2 n6 and C20:4 were significantly (*p* < 0.001) lower, and C20:5 n3 was significantly (*p* < 0.05) higher in the milk from group H than that from group C. The n6/n3, LA/ALA and AA/EPA ratios were significantly (*p* < 0.001) lower in the milk from group H, while the CLAs were unaffected by the treatment.

## 1. Introduction

*Cannabis sativa* L., commonly known as hemp, is a cosmopolitan annual herbaceous plant historically cultivated as a source of textile fiber. In recent years, its seeds and oil have garnered especially great interest as animal feed due to their chemical and nutritional properties [1]. The high content of polyunsaturated fatty acids (PUFA), particularly the n6 series (linoleic acid) and the n3 series (linolenic acid), as well as the presence of aromatic compounds, makes it particularly suitable for improving the nutritional characteristics of animal-derived products [2]. The potential for enhancing the nutritional profile of animal-derived foods through dietary manipulation has already been demonstrated [3]. In particular, several authors [4,5,6] have investigated the impact of incorporating PUFA-rich additives into the diet of lactating ruminants. Their results indicate that an animal diet rich in n3- and n6 fatty acids (FA) can enhance the nutritional profile of milk by increasing its content of beneficial FA, including n3, n6 and conjugated linoleic acid (CLA). It is evident that PUFA in ruminants undergo a biohydrogenation (BH) process, which results in their saturation. However, a considerable amount of PUFAs in the diet evade BH and are directly incorporated into the milk. Furthermore, intermediate compounds of BH serve as substrates for desaturation enzymatic activity in the mammary gland, which is responsible for the highest concentration of CLA in milk [7]. *Cannabis sativa var. sativa* has acquired significant interest due to its low tetrahydrocannabinol (THC) content (<0.2–0.3%), a chemical compound responsible for psychoactive effects. This cultivar is suitable for legal cultivation in Europe for the production of hempseed and hempseed cake to be used as feed ingredients for all animal species [8]. Hemp has been extensively studied as a dietary supplement for ruminants given its fatty acid profile and the presence of bioactive compounds, including cannabidiol and polyphenols, which have been recognized for their beneficial physiological effects [9]. Other research has explored the implications for animal nutrition within the context of a circular economy. This includes investigations into the use of by-products from hemp processing, such as protein cakes [10] or the whole hemp plant after cannabinoid extraction (spent hemp) [11]. The aim of this study was to explore the effects of supplementing goats’ diets with hemp hay on the milk yield, chemical composition, and fatty acid profile. The hypothesis was that the beneficial compounds present in *Cannabis sativa* could improve milk’s nutritional characteristics, and that hemp hay could partially substitute alfalfa hay in goats’ diets. It should be specified that the milk yielded in this trial was used for research purposes only.

## 2. Materials and Methods

### 2.1. Experimental Design

This study was performed at the Azienda Zootecnica Antonio Amato located in Casaletto Spartano (SA, Italy; 832 m.s.l.; 40°09′ N, 15°37′ E; 30–68 mm of rainfall; temperature of 9.8–22 °C) according to the Animal Welfare and Good Clinical Practice (Directive 2010/63/EU) and was approved by the local animal ethics committee (protocol number: PG/2019/0070006). Twenty multiparous Murciana goats (body weight, BW: 49 ± 2 kg; 3rd parity; milk yield of 2250 ± 200 g/head/day) at 60 ± 7 days in milk were randomly allocated into two groups (C: control vs. H: hemp). All subjects had free access to water and to a spontaneous pasture (from 9:00 a.m. to 4:00 p.m.; 12 Ha) comprising plants, shrubs, and trees. After grazing, the goats were individually housed in a 1 × 2 m box and fed 500 g/head/day of a barley and corn meal mixture (BCM, 50/50). In addition, group H was given 250 g/head/day of hemp hay, while group C received the same amount of alfalfa hay. Their BW was measured monthly, while the mixture and hay refusals were weighed daily. The animals were milked twice a day for 4 months (from April to July), and the individual milk yields were recorded daily.

### 2.2. Diet and Feed Analysis

The animals were accompanied in the pasture when grazing to identify the plants most commonly eaten. Pasture samples were collected weekly from four distinct areas of 2.5 m^2^ each and pooled monthly. Four representative samples (1 kg) of the same size, obtained the four different areas, were air-oven-dried at 65 °C. Samples of hays and mixture were collected monthly. All the feeds were milled through a 1 mm screen and analyzed in accordance with the AOAC [12] for dry matter (DM, ID 934.01), crude protein (CP, ID 984.13), ether extract (EE, ID 920.29), and ash (ID: 942.05). The fiber fractions were determined according to Van Soest et al. [13], and the nutritive value (UFL = 1700 kcal of net energy for lactation) was calculated as suggested by the INRA [14]. The determination of the fatty acid (FA) profile was conducted by extracting the milk fat using an ASE (accelerated solvent extractor) with petroleum ether as the solvent. The solvent was evaporated under a N2 stream (Genevac EZ-2, SP Industries, Warminster, PA, USA) at 60 °C. The residual samples (extracted lipids in vials) were weighed before adding 4 mL of 1% H_2_SO_4_ in methanol and held at 50° C overnight. Then, hexane (1 mL of hexane per 20 mg of extracted fat) and 4 mL of NaSO4 (0.47% in H_2_O) were added and vigorously agitated to transfer the methylated fatty acids in the organic phase. The organic phase was collected after centrifugation and analyzed by GC-FID with an Agilent 7820A Gas Chromatograph (Agilent Technologies, Santa Clara, CA, USA). Then, 1 μL was injected at a split ratio of 65:1. A Supelco OMEGAWAX-TM 250 (Sigma-Aldrich, St. Louis, MO, USA) (internal diameter of 30 m × 0.25 mm, film thickness of 0.25 μm) was used, with hydrogen as the carrier at 1.4 mL/min. The oven temperature was set to 50 °C, where it was held for 2 min, then raised to 220 °C at a rate of 4 °C/min, and held for 23 min. Both the injector and the detector temperatures were set to 250 °C. The individual FAs were identified by comparing the retention time of the standard FA methyl ester mixture (Supelco 37 Component FAME Mix, 47,885–U, Nu Check GLC 674). Individual FA methyl esters were expressed as percentages of the total area of eluted FA methyl esters.

### 2.3. Milk Sampling and Analysis

Individual milk samples were collected monthly (4 samplings), divided into two sterile Falcon test tubes, and stored at 4 °C. One aliquot was analyzed for protein, fat, and lactose using a Milko Scan FT+ (Foss, Hilleroed, Denmark), which is standardized for goat milk. The second aliquot was stored at −20 °C and then analyzed for the fatty acid profile, as suggested by Zicarelli et al. [15]. In brief, the total fat was extracted with hexanopropanol and isopropanol (3/2 *v*/*v*) [16] and trans-methylated according to Chouinard et al. [17]. The methyl esters were quantified by GC (ThermoQuest 8000TOP gas chromatograph, Thermo Electron Corporation, Rodano, Milan, Italy) with a flame ionization detector and capillary column (CP-SIL 88 fused silica capillary column, internal diameter of 100 m × 0.25 mm with a 0.2 μm film thickness; Varian, Inc. Walnut Creek, CA, USA) and the following temperature ramp: 70 °C for 4 min → 13 °C/min → 175 °C for 27 min → 3 °C/min → 215 °C for 38 min → 10 °C/min → 70 °C. The temperatures of the injector and detector were 250 °C and 260 °C, respectively. The gas flows were as follows: carrier gas (helium)—1 mL/min; hydrogen—30 mL/min; air—350 mL/min; make-up gas (helium)—45 mL/min. The FA peaks were identified by comparing them with a standard mixture of fatty acid methyl esters (Larodan Fine Chemicals, AB, Limhamnsgårdens Malmö, Sweden). CLA isomers were recognized by comparing the samples’ chromatograms with those of single purified isomers (CLA cis-9, trans-11; CLA trans-10, cis-12; CLA cis-9, trans-11; CLA trans-9, trans-11) (Larodan Fine Chemicals, AB, Limhamnsgårdens Malmö, Sweden).

### 2.4. Statistical Analysis

The data were analyzed using repeated measures ANOVA, and the means were compared using the Tukey’s test (SAS, 2000). The milk data were analyzed with two-way ANOVA for repeated measures using JMP software (version 11, PROC GLM, SAS 2000), in accordance with the following model:

Yijk = μ + Di + Sj + (DS)ij + εijk

where y = the mean of the response variable, μ = the general mean, Di = effect of the dietary treatment (i = 2; C and H), Sj = the sampling effect (j milk = 4; I, II, III, IV), DS = interaction between the dietary treatment and sampling effect, and εijk = the residual error. The means were statistically compared using Tukey’s test. Differences were considered statistically significant at *p* < 0.05.

## 3. Results

In Table 1, the chemical compositions of the feeds are reported. The hemp (HH) and alfalfa hays (AH) showed similar crude protein contents, while HH was characterized by higher ether extract and lower NDF, ADF, and ADL than AH. Thus, its nutritive value was higher (UFL/kg DM 0.77 vs. 0.75, for HH and AH, respectively).

Concerning the feeds’ FA profiles (Table 2), the pasture showed significant differences among the months of collection. In particular, the highest levels of saturated (SFA) and monounsaturated (MUFA) fatty acids were detected in April, while polyunsaturated fatty acids (PUFAs) reached their highest levels in May, mainly for those of the n3 series. The BCM was characterized by a high content of PUFA, mainly represented by the n6 series. Hemp hay showed higher values of SFA and MUFA and lower values of PUFA than alfalfa hay. In addition, AH was characterized by higher contents of both n3 PUFA and n6 PUFA than HH.

No feed refusals were detected; moreover, no difference in body weight was found between the groups throughout the study. The milk yield was significantly higher in the experimental group (*p* < 0.05), while no differences were found in the milks’ chemical compositions (Table 3).

The mean values of the milks’ FAs are depicted in Table 4. Several significant differences were found between the groups. In particular, among the SFAs, C11:0 (undecanoic acid), C12:0 (lauric acid), C13:0 (tridecanoic acid), C14:0 (myristic acid), C15:0 (pentadecylic acid), and C17:0 (margaric acid) were significantly lower, and C16:0 (palmitic acid) and C18:0 (stearic acid) were significantly higher in the milk from group H than in that from group C; among the MUFAs, C14:1 (myristoleic acid) was significantly lower and C16:1 (palmitoleic acid), C17:1 (eptadecenoic acid), C18:1 cis 11, and C18:1 cis 12 (optadecenoic acids) were significantly higher in the milk from group H than in that from group C. Among the PUFAs, C18:2 n6 (linoleic acid, LA) and C20:4 n6 (arachidonic acid, AA) were significantly lower, and C20:5 n3 (EPA) was significantly higher in the milk from group H than that from group C.

**Figure 1:**
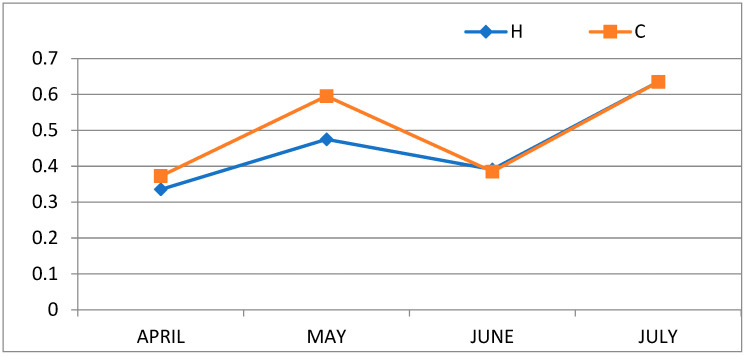
Trends of milk CLAs (g/100 g of fat) throughout the trial.

The FA classes and the nutritional indices are reported in Table 5. The total PUFA and n6/n3, LA/ALA, and AA/EPA ratios were significantly (*p* < 0.001) lower in the milk from group H, while the CLAs were unaffected by the treatment. In both groups, the total CLA increased from April to May, decreased in June, and increased again in July, showing a similar trend to the pasture n3PUFA (Figure 1). The hypercholesterolemic saturated fatty acid (HSFA) and desirable fatty acid (DFA) indices were calculated according to Kumar et al. [18]. The DFAs were significantly (*p* < 0.001) higher in the milk of the supplemented group, whereas no differences were detected for the HSFAs.

## 4. Discussion

The chemical composition of the pasture was similar to that reported by Tudisco et al. [19] in previous research performed in the same area. Concerning the pasture PUFAs, the n3 series showed fluctuating behavior over the months and was higher than the n6 series in all samples, excepted in June according to Lo Presti et al. [20]. In contrast, Meľuchová et al. [21] reported a progressive decrease in C18:3 from May to July and ascribed these differences among the sampling months to the different phenological phases, as suggested by Cabiddu et al. [22]. Hemp hay showed a remarkable chemical composition, with slightly lower crude protein and NDF contents in comparison to the findings of Ran et al. [23], and high content of n3 PUFA, making the plant suitable for animal feeding, as suggested by Bailoni et al. [1]. Indeed, n3 PUFAs in feed has the effect of increasing the levels of beneficial FAs in foods of animal origin, with favorable effects on human health [24,25].

The goats of both groups did not change their body weight throughout the trial. Indeed, according to Tudisco et al. [26], the energy requirements (ER) were satisfied as follows: the ER for maintenance was equal to 0.0365 UFL/kg of metabolic weight (MW/BW of 0.75), while the ER for milk production was equal to 0.41 UFL/kg of fat-corrected milk (FCM, 4% fat). Thus, the total ER was 1.52 UFL (0.67 UFL maintenance + 0.85 UFL milk production), and, considering that the average pasture DM intake in the internal areas of Southern Italy is 20 g/kg BW [27], the deficit of 0.76 UFL was compensated for by the concentrate and hays.

The milk yield was significantly higher in group H (1954 vs. 1731 g/head/day; *p* < 0.05), while no differences were observed in the fat, protein, or lactose. In contrast, other authors [28,29,30,31,32] have reported significant increases in milk fat and protein from using hemp seeds in animal feed.

Concerning SFAs, the milk from the goats of group H showed significantly lower percentages of lauric (C12:0) and myristic (C14:0) acids, both with atherogenic properties. Also, Rapetti et al. [2] found a decrease in lauric acid in the milk from goats fed hemp seed. Interestingly, other authors have observed, in vitro, several potentialities of lauric acid as an antidiabetic enhancing anti-insulin resistance [33,34]. The milk of group H also showed significantly lower percentages of pentadecylic (C15:0) and margaric (C17:0) acids, whose role in reducing the risk of cardiovascular diseases and the incidence of diabetes type 2, as well as the inflammatory state, has been described [5]. Conversely, the percentages of palmitic (C16:0) and stearic (C18:0) acids, the former potentially atherogenic and the latter possibly thrombogenic [26], were significantly higher in the milk of group H. The goats fed hemp hay yielded milk with significantly higher levels of lignoceric acid (C24:0), which is able to promote mitochondrial β-oxidation [35].

Hemp hay feed significantly lowered the milk PUFA and the n6/n3 ratio. It is the responsibility of animal nutritionists to implement feeding strategies that will reduce the ratio of animal-derived food in order to produce products with superior beneficial properties for consumers. A reduction in the dietary n6/n3 ratio has been demonstrated to attenuate the inflammatory response to high-fat meals and to suppress low-grade inflammation, which could contribute to the development of numerous chronic diseases [36]. In addition, in the present trial, this parameter fell within the range suggested as beneficial for human health [37] in both groups, reinforcing the notion that the milk from goats raised on pasture has excellent nutritional characteristics, as observed in previous studies [38]. Moreover, the CLAs, known for their anticarcinogenic effects [39], were remarkable in the milks of both groups. Other authors, using different sources of hemp, have found significant increases in milk’s alfa-linolenic acid and CLAs [2,30,31,32], while Cozma et al. [29] reported a significant increase in the total PUFA in goats fed hempseed oil, however, it did not influence the n3 PUFA content.

The milk of the supplemented group was also found to contain significantly higher levels of DFA, a result that aligns with the observations of Morais et al. [40] in the milk of goats that had been supplemented with Mauritia flexuosa. This index offers evidence of the quality of lipids, which is beneficial and has positive implications for consumer health.

Compared to the control group, the goats fed hemp hay showed significantly lower levels of arachidonic acid (AA, C20:4 n6) but significantly higher levels of EPA (C20:5 n3), as also observed by Mielita et al. [32]. Our findings are inconsistent with those of Rapetti et al. [2], who reported no significant differences in the fatty acid content between goats supplemented with hempseed and a control group. According to Tallima et al. [41], AA plays a fundamental role in promoting cell membrane fluidity and the activity of ion channels mainly in the brain, while EPA contributes to lowering the risk of cardiovascular disease and neuroinflammation, preserving mental health [42]. Nevertheless, despite the biological importance of DHA and ARA in human health, the recommendations regarding specific dietary requirements for these nutrients are inconsistent and, at times, controversial. There is an emerging body of evidence that links AA and its metabolites to the development of chronic diseases, as well as an increase in plasma phospholipids and cholesteryl esters, but only when exceeding the recommended doses [43].

Therefore, from a nutritional point of view, feeding goats hemp hay led to both an improvement and a deterioration of their milk’s fatty acid profile. According to Oh and Hristov [44], this could be affected by the composition of volatile and bioactive compounds, which modulate the rumen metabolism.

Furthermore, the potential use of cannabis sativa as a dietary supplement should be subjected to further investigation from an economic standpoint in animal farming. Indeed, numerous authors [45,46,47] have reported an enhancement of economic efficiency through the utilization of aromatic plants as a feed additive in ruminant diets. This has led to the suggestion that the increase in feed costs was largely offset by the improvement in animal health and performance, both of which increased farm profitability.

## 5. Conclusions

The aim of this research was to evaluate the influence of replacing alfalfa hay with hemp hay in the diets of goats raised on pasture on the milk yield, chemical composition, and fatty acid profile. The goats fed hemp hay showed a significantly higher milk yield without modifications in the chemical composition. Contrasting results from a nutritional point of view were observed for the milk’s FA profile. Indeed, among the atherogenic acids, the lauric and myristic acids decreased, while the palmitic acid increased in the experimental group. Accordingly, the CLAs were unaffected by the treatment, whereas the n6/n3 ratio decreased. This finding is of particular interest in the context of promoting an animal diet that could potentially reduce this ratio, which is of particular importance for human health in the prevention of chronic disease. Therefore, it follows that hemp hay could be a potential feed capable of enhancing milk production. Nevertheless, further research is required to ascertain its influence on milk quality with regard to bioactive and aromatic compounds and to determine the optimal quantity for administration.

## Figures and Tables

**Table 1:** Chemical compositions (g/kg DM) and nutritive values (UFL/kg DM) of feeds.

	Pasture	Barley/Corn Mix	Hemp Hay	Alfalfa Hay
CP	160.2 ± 1.3	100.4 ± 0.9	207.7 ± 2.7	194.5 ± 2.6
EE	22.6 ± 0.3	31.6 ± 2.6	21.2 ± 0.7	16.8 ± 0.8
NDF	481.8 ± 5.1	165.2 ± 1.3	401.5 ± 2.4	413.5 ± 2.3
ADF	429.4 ± 2.51	45.1 ± 1.7	318.4 ± 5.2	344.1 ± 4.9
ADL	46.2 ± 2.9	5.3 ± 2.2	33.4 ± 6.0	51.7 ± 2.7
Ash	72.5 ± 2.2	10.5 ± 1.5	101.9 ± 1.4	99.5 ± 1.9
UFL/kg DM	0.76	1.04	0.78	0.77

CP: crude protein; EE: ether extract; NDF: neutered detergent fiber; ADF: acid detergent fiber; ADL: acid detergent lignin; UFL: feed unity for lactation; DM: dry matter.

**Table 2:** Fatty acid profiles (g/100 g fat) of pasture, barley/corn mixture (BCM), hemp hay (HH), and alfalfa hay (AH).

	Pasture				BCM	HH	AH
	April	May	June	July			
SFA	39.2A	22.3 D	26.4C	34.6B	20.0	39.0	25.3
MUFA	22.8A	11.2C	17.8B	16.4B	21.5	23.5	8.2
PUFA	38.0D	65.5A	55.8B	49.0C	58.5	37.5	66.5
n6 PUFA	16.2C	24.1B	29.9A	10.31D	55.4	8.3	17.1
n3 PUFA	21.3D	40.1A	25.3C	37.9B	1.6	15.6	34.8

SFA: saturated fatty acids; MUFA: monounsaturated fatty acids; PUFA: polyunsaturated fatty acids.

**Table 3:** Body weight (kg); mean milk yield (g/head/day), and chemical composition (%).

	H	C	RMSE
Body weight	49.1	49.2	6.3
Milk yield	1954.0 a	1731.0 b	503.5
Protein	3.26	3.16	0.20
Fat	2.99	3.07	1.74
Lactose	4.15	4.05	0.18

a, b: *p* < 0.05; RMSE: root mean square error.

**Table 4:** Milk fatty acid profile (g/100 g of fat).

Item			Group Effect	Time Effect	G × T	RMSE
	Group H	Group C	*p*	*p*	*p*	
SFA						
C4:0	3.041	3.214	NS	***	NS	0.774
C6:0	1.737	1.824	NS	NS	***	0.190
C8:0	2.421	2.682	NS	***	***	0.364
C10:0	10.80	10.70	NS	***	***	1.063
C11:0	0.089	0.251	***	***	***	0.066
C12:0	3.687	4.658	***	*	***	0.677
C13:0	0.055	0.112	***	***	***	0.022
C14:0	9.585	10.995	***	***	***	0.835
C15:0	0.699	0.822	***	***	***	0.109
C16:0	29.17	27.93	*	NS	NS	2.299
C17:0	0.650	0.860	***	***	***	0.196
C18:0	13.26	11.23	***	***	*	2.085
C20:0	0.186	0.197	NS	**	NS	0.042
C22:0	0.103	0.124	***	***	*	0.022
C24:0	0.039	0.030	***	*	***	0.009
MUFA						
C14:1	0.166	0.294	***	**	**	0.074
C16:1	0.617	0.471	***	***	***	0.072
C17:1	0.024	0.019	**	NS	NS	0.005
C18:1 CIS6	0.118	0.113	NS	***	*	0.023
C18:1 trans 9	0.276	0.315	NS	***	NS	0.134
C18:1 trans 11 (TVA)	1.437	1.320	NS	**	NS	0.267
C18:1 CIS9	17.13	16.89	NS	**	**	1.155
C18:1 CIS10	0.323	0.341	NS	***	*	0.099
C18:1 CIS11	0.199	0.143	***	***	***	0.053
C18:1 CIS12	0.083	0.069	*	***	*	0.022
C22:1	0.016	0.012	NS	NS	NS	0.009
C24:1	0.021	0.027	*	**	NS	0.008
PUFA						
C18:2 TRANS n6	0.116	0.135	NS	***	***	0.046
C18:2 CIS n6 (LA)	1.726	1.980	***	***	***	0.238
C18:3 n6	0.031	0.026	NS	***	NS	0.014
C18:3 n3 (ALA)	0.808	0.792	NS	***	NS	0.149
CLA1	0.239	0.333	***	***	NS	0.064
CLA2	0.219	0.161	***	***	***	0.034
C20:2 n6	0.022	0.037	***	***	***	0.010
C20:3 n6	0.019	0.019	NS	*	NS	0.020
C20:4 n6 (AA)	0.108	0.125	***	***	NS	0.017
C22:2 n6	0.017	0.017	NS	**	NS	0.007
C20:5 n3 (EPA)	0.053	0.044	*	***	**	0.013

* *p* < 0.05; ** *p* < 0.01; *** *p* < 0.001; SFA: saturated fatty acids; MUFA: monounsaturated fatty acids; PUFA: polyunsaturated fatty acids. RMSE: root mean square error. NS: not significant.

**Table 5:** Milk fatty acid classes (g/100 g of fat) and ratios.

Item	Group H	Group C	Group Effect	Time Effect	G × T	RMSE
SFA	75.52	75.64	NS	***	*	1.107
MUFA	20.42	20.04	NS	***	***	1.105
PUFA	3.38	3.67	***	***	***	0.325
n6	2.05	2.34	***	***	***	0.237
n3	0.871	0.837	NS	***	NS	0.151
CLAs	0.459	0.494	NS	***	NS	0.073
PUFA/SFA	0.044	0.048	**	***	***	0.044
n6/n3	2.40	2.98	***	***	**	0.515
LA/ALA	2.17	2.66	***	**	**	0.523
AA/EPA	2.31	3.19	***	***	**	0.837
DFA	37.06	36.01	***	**	NS	0.352
HSFA	42.43	43.01	NS	NS	NS	0.376

SFA: saturated fatty acids; MUFA: monounsaturated fatty acids; PUFA: polyunsaturated fatty acids; CLA: conjugated linoleic acids; LA: linoleic acid; ALA: alpha-linolenic acid; AA: arachidonic acid; EPA: eicosapentaenoic acid; DFA: desirable fatty acid; HSFA: hypercholesterolemic saturated fatty acid. * *p* < 0.05; ** *p* < 0.01; *** *p* < 0.001. RMSE: root mean square error. NS: not significant.

## Data Availability

The datasets generated during and/or analyzed during the current study are available from the corresponding author upon reasonable request.
